# Maternal Characteristics, Mean Arterial Pressure and Serum Markers in Early Prediction of Preeclampsia

**DOI:** 10.1371/journal.pone.0063546

**Published:** 2013-05-22

**Authors:** Sylwia Kuc, Maria P. H. Koster, Arie Franx, Peter C. J. I. Schielen, Gerard H. A. Visser

**Affiliations:** 1 Department of Obstetrics, Wilhelmina Children’s Hospital, University Medical Centre Utrecht, Utrecht, The Netherlands; 2 Laboratory for Infectious Diseases and Screening, National Institute for Public Health and the Environment, Bilthoven, The Netherlands; VU University Medical Center, The Netherlands

## Abstract

**Objectives:**

In a previous study, we have described the predictive value of first-trimester Pregnancy-Associated Plasma Protein-A (PAPP-A), free β-subunit of human Chorionic Gonadotropin (fβ-hCG), Placental Growth Factor (PlGF) and A Disintegrin And Metalloprotease 12 (ADAM12) for early onset preeclampsia (EO-PE; delivery <34 weeks). The objective of the current study was to obtain the predictive value of these serum makers combined with maternal characteristics and first-trimester maternal mean arterial blood pressure (MAP) in a large series of patients, for both EO-PE and late onset PE (LO-PE; delivery ≥ 34 weeks).

**Methods:**

This was a nested case-control study, using stored first-trimester maternal serum from women who developed EO-PE (n = 68) or LO-PE (n = 99), and 500 uncomplicated singleton pregnancies. Maternal characteristics, MAP, and pregnancy outcome were collected for each individual woman and used to calculate prior risks for PE in a multiple logistic regression model. Models containing prior PE risks, serum markers, and MAP were developed for the prediction of EO-PE and LO-PE**.** The model-predicted detection rates (DR) for fixed 10% false-positive rates were calculated for EO-PE and LO-PE with or without the presence of a small-for-gestational age infant (SGA, birth weight <10^th^ centile).

**Results:**

The best prediction model included maternal characteristics, MAP, PAPP-A, ADAM12, and PlGF, with DR of 72% for EO-PE and 49% for LO-PE. Prediction for PE with concomitant SGA was better than for PE alone (92% for EO-PE and 57% for LO-PE).

**Conclusion:**

First-trimester MAP, PAPP-A, ADAM12, and PlGF combined with maternal characteristics and MAP are promising markers in the risk assessment of PE, especially for EO-PE complicated by SGA.

## Introduction

Pre-eclampsia (PE) affects approximately 2% of pregnant women worldwide and is a leading cause of maternal and perinatal morbidity and mortality, in particular when resulting in a delivery before 34 weeks of gestation [1]–[3]. The latter is called early onset PE (EO-PE). It is associated with insufficient placentation and consequently often with severe perinatal morbidity and mortality due to concomitant fetal growth restriction, iatrogenic preterm birth, placental abruption and stillbirth [4]; [5]. The exact pathophysiology of PE remains to be elucidated. Nevertheless it is widely acknowledged that PE is a syndrome that may arise by different pathophysiological pathways in which impaired placentation, maternal constitution and abnormal circulatory and immunological adaptation to pregnancy may play a role. Depending on these pathways PE may occur early in pregnancy with impaired placentation and with fetal growth restriction (‘placental-PE’), or late in pregnancy without fetal growth restriction (‘maternal-PE’) [6]–[8].

First-trimester screening for PE may prove useful to identify high risk patients for subsequent increased surveillance and/or early preventive treatment with low dose aspirin [9]; [10]. Screening with markers of placentation is likely to identify early placental PE, whereas maternal characteristics may be related to both EO- and LO-PE.

In 2010, we performed a nested case-control study in 568 singleton pregnancies at 8^+0^–13^+6^ weeks of gestation, including 88 women who developed EO-PE [11]. In that study we investigated the maternal serum concentrations of serum markers: Pregnancy-Associated Plasma Protein-A (PAPP-A), free β-subunit of human Chorionic Gonadotropin (fβ-hCG), Placental Growth Factor (PlGF), Placental Protein 13 (PP13) and A Disintegrin And Metalloprotease 12 (ADAM12). For a fixed 10% false-positive rate we were able to identify 54% of EO-PE pregnancies. Furthermore, in pregnancies complicated by PE and with an infant small-for-gestational age (SGA, birth weight <10^th^ centile) all serum marker levels were significantly lower as compared to those of pregnancies complicated by PE alone.

Several maternal characteristics are also known to be related to the risk of developing PE. Such factors are nulliparity, higher maternal age, high body mass index (BMI), PE in previous pregnancy, family history of hypertensive pregnancy disorders and chronic hypertension. Also, the mean arterial pressure (MAP) in the first-trimester may provide valuable information [1]; [12]; [13].

It was the aim of this study to examine the performance of first-trimester screening for both EO-PE and LO-PE based on maternal characteristics, first-trimester MAP, and placenta derived serum makers in a new patient population. Prospective studies on markers for PE require large data sets given the low 1–2% incidence of PE. We therefore conducted a nested case-control study using stored first-trimester serum. This way we were able to study 68 EO-PE, 99 LO-PE and 500 controls.

## Materials and Methods

### Ethics Statement

This study approach was approved by the Scientific Ethical Committee of the University Medical Centre of Utrecht (METC Utrecht), the Netherlands (protocol number: 11-002). All participating women in this manuscript have given written informed consent during the first-trimester Down syndrome screening.

### Study Population

This was a nested case-control study derived from a large cohort of women participating in the routine Dutch first-trimester Down syndrome screening between 2007 and 2009. In this context maternal age, sample date, gestational age (GA) at sampling, maternal weight, method of conception, history of diabetes, and smoking status were recorded by a midwife or gynaecologist. As part of the screening, maternal serum concentrations of PAPP-A and fβ–hCG were measured in serum of blood sampled at 9^+0^–13^+6^ weeks GA. Samples were subsequently stored at −80°C. An ultrasound measurement of fetal crown-rump length (CRL) and nuchal translucency (NT) thickness were performed between 11^+4^ and 13^+6^ GA. All ultrasound examinations were performed by certified sonographers using standardized techniques. Where applicable, GA was calculated based on first-trimester CRL measurement at ultrasound examination using the formula of Robinson and Fleming (1975) [14]; otherwise the first day of the last menstrual period was used. Pregnancy outcomes, including chromosomal disorders, date of birth, birthweight and hypertensive pregnancy complications (PE, HELLP syndrome or pregnancy induced hypertension), were collected through self-reporting by the participating women. Six months after the estimated delivery date, a reminder letter was sent to these women to collect missing data. This way, 75% of all pregnancy outcomes could be recorded. For the current study women with a multiple pregnancy, women who delivered before 24 weeks and women who gave birth to a child with a chromosomal abnormality were excluded.

By follow up of self-reported cases of EO-PE we confirmed the diagnosis of EO-PE in 68 pregnancies at the participating hospitals. Moreover, from a larger cohort of women who developed LO-PE we randomly selected 99 cases; 50 cases with an infant appropriate for gestational age at birth and 49 cases with a SGA infant. From all these cases we collected missing data on maternal characteristics i.e. medical history, parity, weight, height, first-trimester mean arterial pressure (MAP) and pregnancy outcome i.e. GA at delivery, birthweight and fetal sex. For the control group 500 women having delivered phenotypically and chromosomally normal neonates at term (37^+0^–42^+0^ weeks) and not having developed any pregnancy complication, were randomly selected from the two biggest ultrasound centres participating in the routine Dutch first-trimester Down syndrome screening program (Universitair Verloskundig Centrum Utrecht and De Poort Leiden). The outcomes of these pregnancies were confirmed in the midwifery practices and missing maternal characteristics and first-trimester MAP were collected.

This was the first time this large cohort of women participating in the routine Dutch first-trimester Down syndrome screening (2007–2009) was used for any research purpose. The data set presented in this study has never been used before in any of our previous studies.

### Outcome Measures

PE was defined according to the criteria of the International Society for the Study of Hypertension in Pregnancy as: gestational hypertension beyond 20 weeks GA in previously normotensive women with a systolic blood pressure ≥ 140 mm Hg and/or diastolic blood pressure ≥ 90 mm Hg on at least two occasions four hours apart, with the presence of proteinuria of ≥ 300 mg in 24-hour collection or at least 2+ by dipstick on a spot urinalysis [2]. EO-PE was defined as PE in pregnancies delivering <34 weeks GA, and LO-PE as PE in pregnancies delivering ≥ 34 weeks. Pregnancy at term was defined as delivery ≥ 37 weeks of GA.

First-trimester blood pressure was collected from the medical records at the hospitals and midwifery practices. It were measured following the standard method of measuring blood pressure in the pregnancy described in the guidelines for gynaecologists and midwives (NVOG: http://www.nvog-documenten.nl and KNOV: http://www.knov.nl/docs). Blood pressure was measured with validated hand devices, calibrated yearly. The pregnant women were in the sitting position for at least 2–3 minutes and preferably their right arm was supported, with the upper arm at heart level. Either a normal or large adult cuff was used depending on the mid-arm circumference. Korotkov V was used to determine the diastolic pressure and the value was noted with a 2 mmHg accuracy. MAP was calculated from the formula *DP +1/3 (SP – DP)*, where DP represents diastolic blood pressure and SP - systolic blood pressure.

Growth charts corrected for gestational age, sex and parity according to the Dutch Perinatal Registry were used to calculate the birthweight z-scores ([15]; (http://www.perinatreg.nl). Weight for GA at the 50^th^ centile was used as the mean of the population and the average standard deviation (SD) was calculated by the formula *(−1 SD +1 SD)/2)*. Subsequently, the z-score was converted into an exact centile for each studied infant. Small-for-gestational age (SGA) was defined as occurring when the birthweight was under the 10^th^ centile.

### Sample Analysis

The concentrations of PlGF and ADAM12 were measured in serum samples thawed once. AutoDELFIA time resolved assays (Perkin Elmer, Turku, Finland) were used to measure all serum marker concentrations. The detection limit (DL) of PlGF was 5.9 pg/mL, all measurements under the DL were discarded (serum samples of 6 controls and 3 cases). For ADAM12 (DL: 6.0 ng/ml), PAPP-A (DL: 5.0 mU/L) and fβ–hCG (DL; 1.5 ng/ml; together with PAPP-A already measured as part of the routine first-trimester Down syndrome screening), none of the measurements was lower than the DL. Before analysis extensive validation was performed for PlGF and ADAM12 assays. Mean intra-assay and inter-assay coefficients of validation for the assays were below 5% at all levels. Due to insufficient stored serum, ADAM12 measurements were not available for 9 controls and 9 cases and PlGF measurements for 12 controls and 14 cases.

### Statistical Analysis

The comparison between PE groups (EO-PE and LO-PE) and controls was made by chi-square test for categorical variables and Mann Whitney-U test for continuous variables, both with *post hoc* Bonferroni correction. The Bonferroni correction was applied to counteract the use of multiple comparisons. Therefore the critical statistical significance was set at p<0.0167.

The following steps described previously by Akolekar *et al.* were undertaken to develop the models for prior risk for EO-PE and LO-PE based on maternal characteristics [1]. 1) The association of various variables (height [19 mean imputations], logarithmically transformed weight [one mean imputation], nulliparity, age, and smoking status with EO-PE and LO-PE was evaluated to establish whether this was a linear or non-linear relationship. If necessary, variables were logarithmically transformed to provide normal distributions. 2) Univariate analysis was done to explore whether the individual maternal characteristics contributed significantly to EO-PE and LO-PE by assessing their odds ratios (ORs) and 95% confidence intervals (CI). 3) Logistic regression analysis with backward stepwise elimination of variables was used to develop the prior risk models for EO-PE and LO-PE. 4) Shrinkage factors (applied to all the parameters in the models to adjust for overfitting) were calculated using the equation [x^2^−(*degrees of freedom*−1)]/x^2^ where x^2^ is the model chi-square derived from the log-likelihood statistic. 5) The patient-specific risks for EO-PE and LO-PE were calculated using formula: odds/(1+ odds), where odds = exp(Y) and Y was derived from the logistic regression analysis.

Serum marker levels and MAP were expressed as multiples of the gestation-specific normal medians (MoMs). Normal medians were obtained by regression analysis of the median concentration for each completed gestational week in controls, weighted for the number of women tested. If MoMs were significantly correlated with maternal weight (PAPP-A, fβ–hCG, ADAM12 and MAP), the observed MoM value was divided by the expected value for the maternal weight based on regression analysis in the controls. When MoMs were significantly different between smoking and non-smoking women a correction factor for smoking was applied (PlGF). The distributions of serum marker MoMs and MAP MoMs were made Gaussian by logarithmical transformation (log_10_). Likelihood ratio’s (LR) were calculated for all markers and subsequently multiplied with the prior risk for EO-PE and LO-PE to calculate posterior risks. Correlation coefficients for the markers were calculated using the log_10_ (all serum markers) and normal (MAP) concentrations/values and gestation, after excluding outliers exceeding 3 standard deviations from the median.

The posterior risks for EO-PE and LO-PE were calculated using different combinations of variables: (1) prior risks based on maternal characteristics, serum markers, and MAP separately, (2) prior risks combined with serum markers or MAP, and (3) prior risk combined with serum markers and MAP. The distributions of the prior and posterior risks were then used to calculate detection rates (DR) and false positive rates (FPRs) at different risk cut-offs by receiver operating characteristic (ROC) curves analysis. Moreover, the EO-PE and LO-PE models were used for prediction of PE cases complicated by SGA (EO-PE with SGA and LO-PE with SGA).

Statistical analyses were performed using SPSS (release 20.0; Chicago, IL) and SAS software package (release 9.2; SAS Institute, Cary, NC, USA).

## Results

Baseline characteristics of the study population are shown in *Table 1*. Women who developed PE had higher BMI (EO-PE p<0.0001; LO-PE p = 0.005), were more often smokers (EO-PE p = 0.008), and more often had a history of hypertensive pregnancy disorders compared to controls (EO-PE p = 0.009; LO-PE p<0.0001). Furthermore, there were more nulliparous women among the cases (both EO-PE and LO-PE p<0.0001).

**Table 1:** Study population baseline characteristics in control and PE pregnancies. Values are presented as median (IQR) or number (%).

Characteristics	Controls	EO-PE	LO-PE
	n = 500	n = 68	n = 99
Maternal age (y)	33 (30–35)	34 (30–37)	33 (30–36)
Maternal weight (kg)	65.5 (60.0–73.0)	70.0 (62.0–81.5)*	67.5 (62.0–75.0)
Maternal BMI (kg/m2)	22.8 (20.7–24.8)	24.7 (21.9–29.3)*	23.7 (21.3–26.5)*
Nulliparity	233 (46.6)	55 (80.9)*	72 (72.7)*
Smoking	21 (4.2)	8 (11.8)*	6 (6.1)
Assisted reproduction	0 (0)	3 (4.4)	8 (8.1)
Gestation at sampling (days)	88 (84–91)	85 (76–89)*	85 (79–89)*
History of hypertensive pregnancy disorders	4 (0.8)	4 (5.9)*	10 (10.1)*
Gestation at birth (wk)	40 (39–41)	31 (30–32)*	37 (36–39)*
Birthweight (gr)	3544 (3243–3800)	1300 (1045–1609)*	2650 (2130–3110)*
Birthweight centile	57.0 (33.1–78.4)	25.0 (13.4–50.4)*	13.8 (3.8–46.0)*
Sex, n male (%)	244 (48.8)	34 (49.7)	53 (53.5)

A Pearson’s chi square test and Mann-Whitney U test, both with *post hoc* Bonferroni correction were used for statistical analysis. Adjusted significance value p<0.016 (*). EO-PE: early-onset preeclampsia; LO-PE: late-onset preeclampsia; IQR: interquartile range; BMI: body mass index.

Multiple regression analysis in the control group resulted in the following median equations for placental marker and MAP:

Log_10_ expected PAPP-A = (0.52405+0.03234×GA at sampling in days)/(−0.19564+76.2423/maternal weight in kg).

Log_10_ expected fβ-hCG = (2.64423−0.01236×GA at sampling in days)/(0.2947−0.00429×maternal weight in kg).

Log_10_ expected PlGF = (0.4999+0.0118×GA at sampling in days)/1.33 [if smoking]; or (0.4999+0.0118×GA at sampling in days)/0.991 [if no smoking].

Log_10_ expected ADAM12 = (−493.422+10.6898×GA at sampling in days)/(0.29637−0.00442×maternal weight in kg).

Expected MAP = 101.052−1321.65/maternal weight in kg.

*Table 2* shows the distribution of median MoM values of all markers in controls and PE groups (with and without SGA). In case of EO-PE, PlGF MoM was significantly lower (0.94 MoM, p = 0.014) and MAP was significantly higher (1.04 MoM, p<0.0001) compared to controls. Lower PlGF MoM and higher MAP MoM were also found in LO-PE pregnancies, however the difference in PlGF MoM did not reach statistical significance (MAP –1.05 MoM, p<0.0001; PlGF –0.90, p = 0.024). In EO-PE pregnancies complicated by SGA there was a clear tendency for all serum markers to be even more reduced. MAP was again statistically significantly increased in the EO-PE SGA group as compared to controls (1.04 MoM, p = 0.01).

**Table 2:** Median MoM (IQR) of marker concentrations in control and PE groups.

Variables	Controls		EO-PE				LO-PE			
			All casesn = 68		With SGA infantn = 13		All casesn = 99		With SGA infantn = 49	
	n	MoM	n	MoM	n	MoM	n	MoM	n	MoM
**PAPP-A**	500	1.01 (0.70–1.48)	68	0.89 (0.53–1.45)	13	0.56 (0.35–1.22)	99	1.04 (0.62–1.49)	49	0.95 (0.54–1.44)
**Free ** ***β*** **–hCG**	500	0.99 (0.70–1.44)	68	0.92 (0.56–1.57)	13	0.88 (0.57–1.06)	99	1.04 (0.70–1.46)	49	1.04 (0.66–1.38)
**ADAM12**	491	1.00 (0.80–1.20)	63	0.93 (0.68–1.15)	12	0.68 (0.57–1.03)*	95	1.02 (0.85–1.28)	48	0.99 (0.81–1.19)
**PlGF**	482	1.00 (0.81–1.25)	59	0.94 (0.68–1.16)*	12	0.83 (0.42–1.15)	91	0.90 (0.65–1.22)	47	0.81 (0.56–1.15)
**MAP**	497	0.99 (0.93–1.05)	58	1.08 (1.02–1.17)*	13	1.04 (1.00–1.12)*	85	1.05 (1.00–1.14)*	37	1.01 (0.98–1.10)*

A Mann-Whitney U test, with *post hoc* Bonferroni correction were used for statistical analysis. Adjusted significance value p<0.016 (*).

MoM: multiple of the median; IQR: interquartile range; PAPP-A: Pregnancy-Associated Plasma Protein-A; fβ–hCG: free β–human Chorionic Gonadotropin; ADAM12: A Disintegrin And Metalloprotease 12; PlGF: Placental Growth Factor; MAP: Mean Arterial Pressure; EO-PE: early-onset preeclampsia; LO-PE: late-onset preeclampsia.

**Figure 1:**
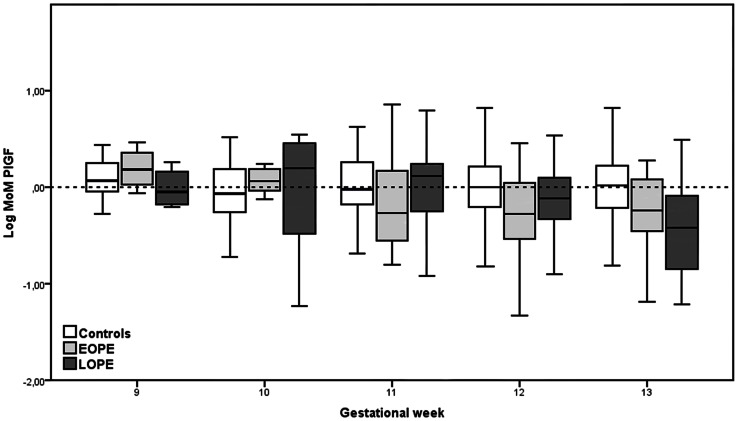
Distribution of logarithmically transformed MoM PlGF through gestation weeks 9^+0^–13^+6^. MoM: multiple of the median.

We studied the distribution of all markers throughout the first-trimester. The difference in median MoM PlGF between both PE groups and controls appeared to be more distinctive ≥11 weeks of gestation (*Figure 1*). Corresponding median MoM values of PlGF ≥11 weeks were substantially lower: 0.77 (p<0.0001) and 0.89 (p = 0.005) for EO-PE and LO-PE, respectively. This trend was only found for PlGF, since there were no differences in the MoM distributions of the other markers (data not shown).

Patient specific prior risks for EO-PE and LO-PE (with and without SGA) were based on maternal age, weight, height, parity, and smoking status (*Table 3*). The shrinkage coefficients for EO-PE and LO-PE models were both 0.94. The analysis resulted in the following equations:

**Table 3:** Multivariate logistic regression analysis of the factors defining the prior risk for the prediction of EO-PE and LO-PE by maternal characteristics.

Independent variable	EO-PE			LO-PE		
	OR	95% CI	*p*	OR	95% CI	*p*
Age	1.095	1.006–1.193	0.036	1.075	1.008–1.147	0.026
Ln Weight	175.634	22.921–1345.844	<0.0001	11.437	2.619–49–949	<0.0001
Height (cm)	0.881	0.835–0.930	<0.0001	–	–	–
Nulliparity	7.1	3.106–16.229	<0.0001	3.981	2.31–6.858	<0.0001
Smoking	4.210	1.543–11.487	0.005	–	–	–

OR: odd ratios; CI: confidence interval; *p*: significance value; Ln: natural logarithm; EO-PE: early onset preeclampsia; LO-PE: late onset preeclampsia.

Prior risk EO-PE = −6.790–0.119×maternal height (cm) +4.8565×Ln maternal weight +1.845×nulliparity [1] +0.086×maternal age (years) +1.353×smoking [1].

Prior risk LO-PE = −14.374+2.300×Ln maternal weight +1.303×nulliparity [1] +0.068×maternal age (years).

**Figure 2:**
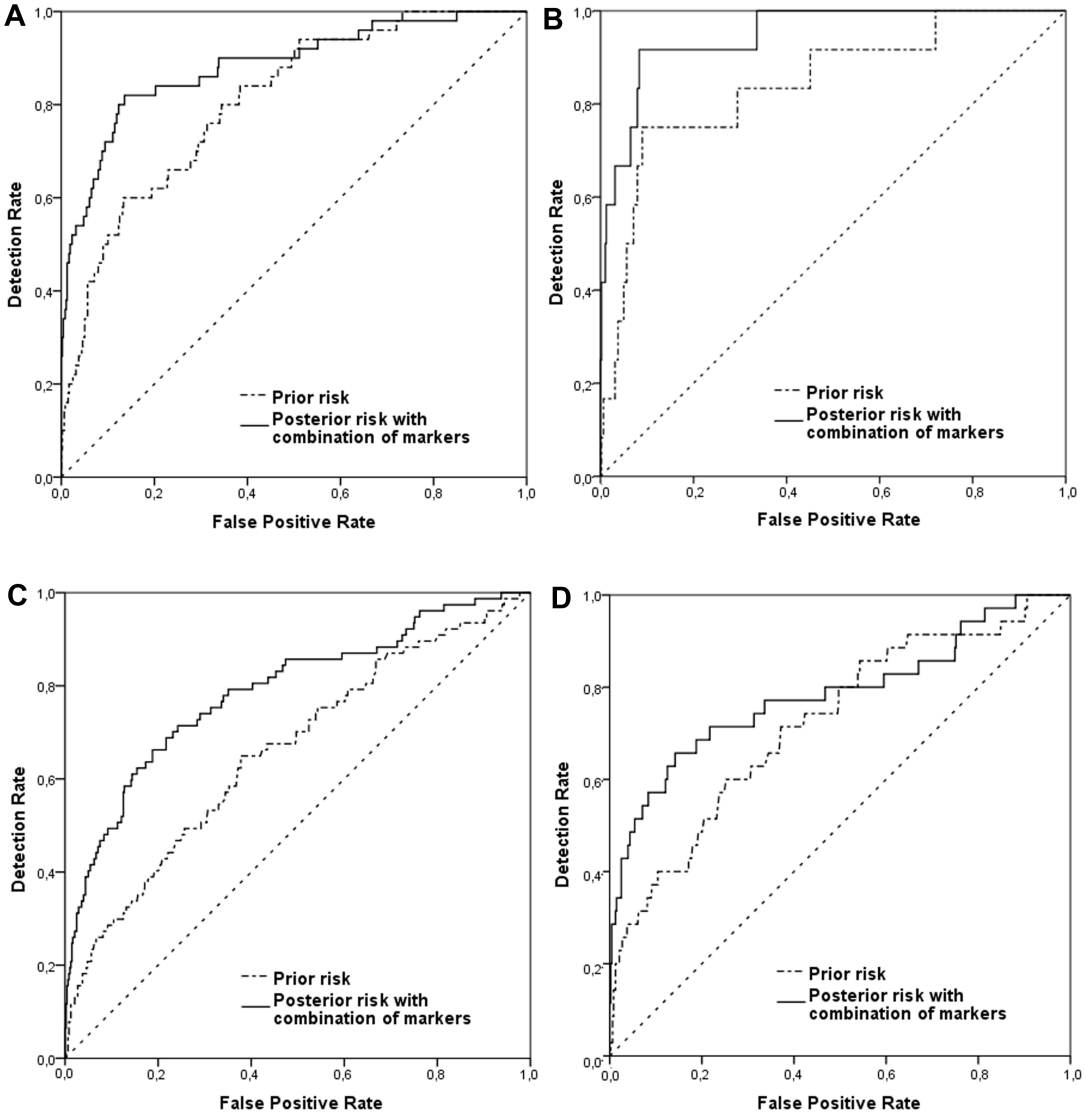
Receiver operating characteristic curves (ROCs) with prediction models for a) EO-PE, b) EO-PE with SGA, c) LO-PE, d) LO-PE with SGA. (- - - -) Prior risk of preeclampsia containing maternal characteristics, (_____) prior risk for preeclampsia combined with all markers: Pregnancy-Associated Plasma Protein-A (PAPP-A), A Disintegrin And Metalloproteinase 12 (ADAM12), Placental Growth Factor (PlGF) and Mean Arterial Pressure (MAP).

Model-predicted detection rates for EO-PE and LO-PE (with or without SGA), for fixed false positive rates are shown in *Table 4*. The detection rates based on maternal characteristics only were 56% and 31% for EO-PE and LO-PE, respectively. The best predictive model contained the combination of markers: maternal characteristics, MAP, PAPP-A, ADAM12 and PlGF (EO-PE: DR = 72% for 10% false positive rate, area under the curve [AUC] = 0.88). The DRs for SGA groups were even more pronounced, particularly in case of EO-PE. The highest DR (92%) for EO-PE with SGA was obtained by the same combination of markers: maternal characteristics, MAP, PAPP-A, ADAM12 and PlGF, AUC = 0.95 (*Figure 2*).

**Table 4:** Model predicted early preeclampsia detection rate (95% CI) for FPR of 5 and 10% with PAPP-A, fβ–hCG, ADAM12, PlGF and MAP in control and preeclampsia groups.

Screening test	Detection rate (95% confidence interval) for fixed FPR								
	EO-PE				LO-PE				
	Alln = 68		with SGA infantn = 13		All n = 99		with SGA infantn = 49		
	5%	10%	5%	10%	5%	10%	5%		10%
Maternal characteristics	40 (29–52)	56 (44–67)	39 (18–65)	69 (42–87)	22 (16–32)	31 (23–41)	27 (16–30)		41 (28–55)
PAPP-A	12 (6–22)	19 (12–31)	31 (13–58)	46 (23–71)	8 (4–15)	14 (9–22)	8 (3–19)		16 (9–29)
Free *β*–hCG	7 (3–16)	21 (13–32)	8 (2–34)	15 (4–43)	6 (3–13)	14 (9–22)	4 (1–14)		12 (6–24)
ADAM12	13 (7–23)	16 (9–27)	25 (9–54)	33 (14–61)	11 (6–18)	18 (12–27)	10 (5–22)		17 (9–30)
PlGF	17 (10–29)	25 (16–38)	25 (9–54)	33 (14–61)	15 (9–24)	25 (17–35)	23 (14–37)		36 (24–51)
MAP	31 (21–44)	40 (28–53)	15 (5–43)	31 (13–58)	27 (19–37)	31 (22–41)	19 (10–34)		19 (10–34)
PAPP-A and PlGF	17 (10–29)	32 (22–45)	33 (14–61)	42 (19–68)	18 (11–27)	24 (17–34)	28 (17–42)		36 (24–51)
PAPPA, ADAM12 and PlGF	20 (12–32)	31 (20–43)	42 (19–68)	42 (19–68)	19 (12–28)	30 (21–40)	28 (17–42)		40 (28–55)
Maternal characteristics plus									
PAPP-A	47 (36–59)	62 (50–72)	69 (42–87)	69 (42–87)	23 (16–32)	34 (26–44)	29 (18–42)	41 (28–55)	
ADAM12	40 (29–52)	60 (48–71)	58 (32–81)	75 (46–91)	19 (12–28)	31 (22–40)	21 (12–34)	38 (25–52)	
PlGF	51 (38–63)	58 (45–69)	67 (39–86)	75 (46–91)	22 (15–32)	35 (26–45)	34 (22–48)	53 (39–67)	
MAP	50 (37–62)	64 (51–75)	39 (18–65)	62 (35–82)	27 (19–37)	45 (35–55)	24 (13–40)	41 (26–57)	
Maternal characteristics plus combination of markers									
MAP and PAPP-A	53 (41–66)	71 (58–81)	69 (42–87)	69 (42–87)	32 (23–42)	46 (36–56)	32 (20–49)	41 (26–57)	
MAP and PlGF	54 (40–67)	68 (54–79)	67 (39–86)	75 (46–91)	35 (25–46)	56 (45–66)	46 (30–62)	63 (46–77)	
MAP, PAPP-A and PlGF	54 (40–67)	70 (57–81)	50 (25–75)	83 (55–95)	38 (28–49)	52 (41–63)	49 (33–65)	60 (43–74)	
MAP, PAPPA, ADAM12 and PlGF	56 (42–69)	72 (59–83)	67 (39–86)	92 (64–98)	40 (30–51)	49 (38–60)	49 (33–65)	57 (41–72)	

FPR: false positive rate; PAPP-A: Pregnancy-Associated Plasma Protein-A; fβ–hCG: free β–human Chorionic Gonadotropin; ADAM12: A Disintegrin And Metalloprotease 12; PlGF: Placental Growth Factor; MAP: Mean Arterial Pressure; EO-PE: early-onset preeclampsia; LO-PE: late-onset preeclampsia; SGA: small-for-gestational age.

## Discussion

This large retrospective study, showed that serum markers, combined with maternal characteristics such as age, weight, height, nulliparity, smoking status and first-trimester MAP, are a powerful tool to predict PE in the first-trimester. Prediction was better for EO-PE than for LO-PE and for PE complicated by SGA (both early and late).

In 2010 we reported on the results of a combination of three placenta derived serum markers (PAPP-A, PlGF and PP13) and calculated a detection rate for EO-PE of 55% at 10% FPR [11]. In the present study inclusion of maternal characteristics and first-trimester MAP resulted in a higher detection rate (72%), which is in line with other publications [3]; [16]. This seems logical given the different pathways that may result in PE. Serum markers are related to placentation, MAP is related to the maternal vascular adaptation and maternal characteristics determine the susceptibility of the mother [7]; [8]; [17]; [18].

In the current study the DR of the combined placental markers for EO-PE was lower than in our previous study (31% and 55% at 10% FPR, respectively). This might be due to differences in the laboratory assays (ADAM12 and PlGF) and to the absence of the marker PP13, which is commercially unavailable at this moment.

PlGF is a placenta derived pro-angiogenic factor and its concentrations increase throughout pregnancy [19]; [20]. In pregnancies destined to develop PE, concentrations of PlGF are significantly lower compared to healthy controls [11]; [19]; [21]. We found that <11^+0^ weeks of GA MoM values of PE cases were comparable to MoM values of controls. Starting from 11^+0^ weeks onwards, MoM values of PlGF appeared to be significantly lower in PE cases, more dominantly in EO-PE than in LO-PE cases. A systematic literature search resulted in eight studies in which PlGF was measured in the first-trimester of pregnancy [1]; [11]–[13]; [21]–[24]. Six out of the eight studies measured PlGF from 11^+0^ weeks onwards and median MoM values in EO-PE cases varied between 0.59 and 0.69 [1]; [12]; [13]; [21]; [22]; [24]. In the other two studies measurements were carried out from 8^+0^ weeks onwards. The median MoM values in case of EO-PE in these two studies were 0.73 and 0.86, respectively [11]; [23]. Based on these publications and on our data it has to be concluded that signs of impaired placentation should be investigated after 11 weeks GA. We are currently conducting a study to investigate the longitudinal course of several placenta derived serum markers during the first-trimester [20]. Results from this study might provide more insight in the predictive value of PlGF throughout the first-trimester.

Maternal characteristics based on routine medical information were the most informative predictors for EO-PE. For LO-PE maternal characteristics, MAP and serum markers were of equal importance. These data are in agreement with those of Poon et al [13]. Others, however, have found that maternal characteristics were better in case of LO-PE [25]. In our analysis we found that maternal smoking was independently associated with EO-PE. This was an unexpected and most likely a chance finding, given the literature on a possible preventive effect of smoking on PE [26]–[28].

Identification of PE with SGA was better than for PE without SGA, both for EO-PE and LO-PE. This was due to a better predictive value of both maternal characteristics and serum makers, but not of MAP. The relationship of SGA with serum markers seems logical given the fact that these markers are related to early placentation. Also in our previous study in EO-PE we found lower placental markers in PE complicated by SGA [11]. Surprisingly, placental serum markers of the 10% of SGA infants in the control group were similar to those of appropriate-for-gestational age infants (data not shown). This implies that SGA with PE after 34 weeks constitutes a different entity, with signs of impaired early placentation, although not as evident as SGA with EO-PE. In this context it is important to note that late PE consisted of women delivered after 34 weeks and PE at 34–37 weeks is still related to SGA, although less than at earlier gestation [29]–[31]. SGA at term, in the absence of PE, is apparently not related to early-impaired placental development.

First-trimester MAP appears to be one of the most important predictors of PE. A considerable shortcoming of our study may be the fact that this parameter, as the rest of maternal characteristics, was derived from the medical records at the hospitals and midwifery practices in the retrospective manner. However, in the Netherlands blood pressure in pregnancy is measured following the standard method described in the guidelines for gynaecologists and midwives. Therefore we strongly believe that MAP provided here is enough accurate to be able to draw the conclusions from. Standard use of accurate automatic devices might increase its prognostic value in the future.

In this study we did not measure first-trimester uterine artery Doppler. Uterine artery Doppler is known to be one of the best first-trimester PE markers [3]. Addition of this marker to our prediction model may well increase the detection of PE even more. However, uterine artery Doppler measurements should preferably be measured between 11 and 13 weeks of GA and women in the Netherlands do not routinely receive an ultrasound examination at that time. However, we have shown that even without the measurement of uterine artery Doppler detection rates for PE are quite high. The addition of more placenta derived serum markers, such as PP13, may also increase detection rates [3]; [20].

Currently there is emerging evidence that early risk assessment for PE could play an important role in the prevention of PE and subsequent adverse pregnancy outcome. Low-dose aspirin is thought to improve the placental vasculature and therefore reduce the risk for PE. A recent meta-analysis has shown that the prophylactic use of aspirin, started before 16 weeks of GA, may reduce the risk of EO-PE up to 50% and even 90% in case of severe PE, with a 55% reduction in early fetal growth restriction [9]; [10]; [32]. Limited data on LO-PE have not (yet) shown beneficial effects of aspirin, emphasizing differences in aetiology [5]; [7]; [33]. Early identification of PE may also prove useful in differentiation prenatal care, with increased surveillance in high risk women and less intensive surveillance in low-risk women.

In conclusion, we developed a prediction model of PE using a combination of placenta derived markers PAPP-A, ADAM12 and PlGF and first-trimester MAP and maternal characteristic. External validation of this model is needed to confirm that it is a powerful tool for the early prediction of women at risk to develop PE, in particular to predict cases of EO-PE complicated by SGA. Addition of first-trimester uterine Doppler is likely to further improve the model.

## References

[1] Akolekar R, Syngelaki A, Sarquis R, Zvanca M, Nicolaides KH (2011) Prediction of early, intermediate and late pre-eclampsia from maternal factors, biophysical and biochemical markers at 11–13 weeks. Prenat Diagn 31: 66–74. 21210481 10.1002/pd.2660

[2] Brown MA, Lindheimer MD, de Swiet M, Van Assche A, Moutquin JM (2001) The classification and diagnosis of the hypertensive disorders of pregnancy: statement from the International Society for the Study of Hypertension in Pregnancy (ISSHP). Hypertens Pregnancy 20: 9–14. 10.1081/PRG-100104165 12044323

[3] Kuc S, Wortelboer EJ, van Rijn BB, Franx A, Visser GH, et al (2011) Evaluation of 7 serum biomarkers and uterine artery Doppler ultrasound for first-trimester prediction of preeclampsia: a systematic review. Obstet Gynecol Surv 66: 225–239. 21756405 10.1097/OGX.0b013e3182227027

[4] Roberts JM, Pearson GD, Cutler JA, Lindheimer MD (2003) Summary of the NHLBI Working Group on Research on Hypertension During Pregnancy. Hypertens Pregnancy 22: 109–127. 12908996 10.1081/PRG-120016792

[5] Sibai B, Dekker G, Kupferminc M (2005) Pre-eclampsia. Lancet 365: 785–799. 15733721 10.1016/S0140-6736(05)17987-2

[6] Huppertz B (2008) Placental origins of preeclampsia: challenging the current hypothesis. Hypertension 51: 970–975. 18259009 10.1161/HYPERTENSIONAHA.107.107607

[7] Redman CW, Sargent IL (2005) Latest advances in understanding preeclampsia. Science 308: 1592–1594. 15947178 10.1126/science.1111726

[8] Steegers EA, von Dadelszen P, Duvekot JJ, Pijnenborg R (2010) Pre-eclampsia. Lancet 376: 631–644. 20598363 10.1016/S0140-6736(10)60279-6

[9] Bujold E, Morency AM, Roberge S, Lacasse Y, Forest JC, et al (2009) Acetylsalicylic acid for the prevention of preeclampsia and intra-uterine growth restriction in women with abnormal uterine artery Doppler: a systematic review and meta-analysis. J Obstet Gynaecol Can 31: 818–826. 19941706 10.1016/S1701-2163(16)34300-6

[10] Bujold E, Roberge S, Lacasse Y, Bureau M, Audibert F, et al (2010) Prevention of preeclampsia and intrauterine growth restriction with aspirin started in early pregnancy: a meta-analysis. Obstet Gynecol 116: 402–414. 20664402 10.1097/AOG.0b013e3181e9322a

[11] Wortelboer EJ, Koster MP, Cuckle HS, Stoutenbeek PH, Schielen PC, et al (2010) First-trimester placental protein 13 and placental growth factor: markers for identification of women destined to develop early-onset pre-eclampsia. BJOG 117: 1384–1389. 20840693 10.1111/j.1471-0528.2010.02690.x

[12] Foidart JM, Munaut C, Chantraine F, Akolekar R, Nicolaides KH (2010) Maternal plasma soluble endoglin at 11–13 weeks’ gestation in pre-eclampsia. Ultrasound Obstet Gynecol 35: 680–687. 20205159 10.1002/uog.7621

[13] Poon LC, Akolekar R, Lachmann R, Beta J, Nicolaides KH (2010) Hypertensive disorders in pregnancy: screening by biophysical and biochemical markers at 11–13 weeks. Ultrasound Obstet Gynecol 35: 662–670. 20232288 10.1002/uog.7628

[14] Robinson HP, Fleming JE (1975) A critical evaluation of sonar “crown-rump length” measurements. Br J Obstet Gynaecol 82: 702–710. 1182090 10.1111/j.1471-0528.1975.tb00710.x

[15] Visser GH, Eilers PH, Elferink-Stinkens PM, Merkus HM, Wit JM (2009) New Dutch reference curves for birthweight by gestational age. Early Hum Dev 85: 737–744. 19914013 10.1016/j.earlhumdev.2009.09.008

[16] Cnossen JS, Vollebregt KC, de Vrieze N, ter Riet G, Mol BW, et al (2008) Accuracy of mean arterial pressure and blood pressure measurements in predicting pre-eclampsia: systematic review and meta-analysis. BMJ 336: 1117–1120. 18480117 10.1136/bmj.39540.522049.BE PMC2386627

[17] Roberts JM (1998) Endothelial dysfunction in preeclampsia. Semin Reprod Endocrinol 16: 5–15. 9654603 10.1055/s-2007-1016248

[18] Visser N, van Rijn BB, Rijkers GT, Franx A, Bruinse HW (2007) Inflammatory changes in preeclampsia: current understanding of the maternal innate and adaptive immune response. Obstet Gynecol Surv 62: 191–201. 17306041 10.1097/01.ogx.0000256779.06275.c4

[19] Taylor RN, Grimwood J, Taylor RS, McMaster MT, Fisher SJ, et al (2003) Longitudinal serum concentrations of placental growth factor: evidence for abnormal placental angiogenesis in pathologic pregnancies. Am J Obstet Gynecol 188: 177–182. 12548214 10.1067/mob.2003.111

[20] Wortelboer EJ, Koster MP, Kuc S, Eijkemans MJ, Bilardo CM, et al (2011) Longitudinal trends in fetoplacental biochemical markers, uterine artery pulsatility index and maternal blood pressure during the first trimester of pregnancy. Ultrasound Obstet Gynecol 38: 383–388. 21520474 10.1002/uog.9029

[21] Akolekar R, Zaragoza E, Poon LC, Pepes S, Nicolaides KH (2008) Maternal serum placental growth factor at 11+0 to 13+6 weeks of gestation in the prediction of pre-eclampsia. Ultrasound Obstet Gynecol 32: 732–739. 18956425 10.1002/uog.6244

[22] Akolekar R, de Cruz J, Foidart JM, Munaut C, Nicolaides KH (2010) Maternal plasma soluble fms-like tyrosine kinase-1 and free vascular endothelial growth factor at 11 to 13 weeks of gestation in preeclampsia. Prenat Diagn 30: 191–197. 20101671 10.1002/pd.2433

[23] Vandenberghe G, Mensink I, Twisk JW, Blankenstein MA, Heijboer AC, et al (2011) First trimester screening for intra-uterine growth restriction and early-onset pre-eclampsia. Prenat Diagn 31: 955–961. 21717483 10.1002/pd.2807

[24] Yu J, Shixia CZ, Wu Y, Duan T (2011) Inhibin A, activin A, placental growth factor and uterine artery Doppler pulsatility index in the prediction of pre-eclampsia. Ultrasound Obstet Gynecol 37: 528–533. 20737451 10.1002/uog.8800

[25] Llurba E, Carreras E, Gratacos E, Juan M, Astor J, et al. (2009) Maternal history and uterine artery Doppler in the assessment of risk for development of early- and late-onset preeclampsia and intrauterine growth restriction. Obstet Gynecol Int. doi: 10.1155/2009/275613. 10.1155/2009/275613 PMC2778944 19936122

[26] Kahn SR, Almeida ND, McNamara H, Koren G, Genest J Jr, et al.. (2011) Smoking in preeclamptic women is associated with higher birthweight for gestational age and lower soluble fms-like tyrosine kinase-1 levels: a nested case control study. BMC Pregnancy Childbirth doi: 10.1186/1471–2393–11–91. 10.1186/1471-2393-11-91 PMC3248362 22074109

[27] Lindqvist PG, Marsal K (1999) Moderate smoking during pregnancy is associated with a reduced risk of preeclampsia. Acta Obstet Gynecol Scand 78: 693–697. 10468061

[28] North RA, Taylor R, Li Zhou R, Schellenberg JC (2000) The relationship of smoking, preeclampsia, and secretory component. Am J Obstet Gynecol 183: 136–139. 10920321 10.1067/mob.2000.105745

[29] Baschat AA (2004) Pathophysiology of fetal growth restriction: implications for diagnosis and surveillance. Obstet Gynecol Surv 59: 617–627. 15277896 10.1097/01.ogx.0000133943.54530.76

[30] Dieber-Rotheneder M, Beganovic S, Desoye G, Lang U, Cervar-Zivkovic M (2012) Complex expression changes of the placental endothelin system in early and late onset preeclampsia, fetal growth restriction and gestational diabetes. Life Sci 91: 710–715. 22580289 10.1016/j.lfs.2012.04.040

[31] Roberts JM, Escudero C (2012) The placenta in preeclampsia. Pregnancy Hypertens 2: 72–83. 22745921 10.1016/j.preghy.2012.01.001 PMC3381433

[32] Roberge S, Giguere Y, Villa P, Nicolaides K, Vainio M, et al.. (2012) Early administration of low-dose aspirin for the prevention of severe and mild preeclampsia: a systematic review and meta-analysis. Am J Perinatol doi: 10.1055/s-0032–1310527. 10.1055/s-0032-1310527 22495898

[33] Borzychowski AM, Sargent IL, Redman CW (2006) Inflammation and pre-eclampsia. Semin Fetal Neonatal Med 11: 309–316. 16828580 10.1016/j.siny.2006.04.001

